# Modifying ankle foot orthosis stiffness in patients with calf muscle weakness: gait responses on group and individual level

**DOI:** 10.1186/s12984-019-0600-2

**Published:** 2019-10-17

**Authors:** Niels F. J. Waterval, Frans Nollet, Jaap Harlaar, Merel-Anne Brehm

**Affiliations:** 1 Amsterdam UMC, University of Amsterdam, Department of Rehabilitation, Amsterdam Movement Sciences, Meibergdreef 9, Amsterdam, the Netherlands; 2 Amsterdam UMC, Vrije Universiteit Amsterdam, Department of Rehabilitation Medicine, Amsterdam Movement Sciences, De Boelelaan, 1117 Amsterdam, the Netherlands; 3 Department of Biomechanical Engineering, Delft University of Technology, Delft, the Netherlands

**Keywords:** Ankle foot orthosis, Neuromuscular disease, Stiffness, Gait, Walking energy cost, Muscle weakness, Rehabilitation

## Abstract

**Background:**

To improve gait, persons with calf muscle weakness can be provided with a dorsal leaf spring ankle foot orthosis (DLS-AFO). These AFOs can store energy during stance and return this energy during push-off, which, in turn, reduces walking energy cost. Simulations indicate that the effect of the DLS-AFO on walking energy cost and gait biomechanics depends on its stiffness and on patient characteristics. We therefore studied the effect of varying DLS-AFO stiffness on reducing walking energy cost, and improving gait biomechanics and AFO generated power in persons with non-spastic calf muscle weakness, and whether the optimal AFO stiffness for maximally reducing walking energy cost varies between persons.

**Methods:**

Thirty-seven individuals with neuromuscular disorders and non-spastic calf muscle weakness were included. Participants were provided with a DLS-AFO of which the stiffness could be varied. For 5 stiffness configurations (ranging from 2.8 to 6.6 Nm/degree), walking energy cost (J/kg/m) was assessed using a 6-min comfortable walk test. Selected gait parameters, e.g. maximal dorsiflexion angle, ankle power, knee angle, knee moment and AFO generated power, were derived from 3D gait analysis.

**Results:**

On group level, no significant effect of DLS-AFO stiffness on reducing walking energy cost was found (*p* = 0.059, largest difference: 0.14 J/kg/m). The AFO stiffness that reduced energy cost the most varied between persons. The difference in energy cost between the least and most efficient AFO stiffness was on average 10.7%. Regarding gait biomechanics, increasing AFO stiffness significantly decreased maximal ankle dorsiflexion angle (− 1.1 ± 0.1 degrees per 1 Nm/degree, *p* < 0.001) and peak ankle power (− 0.09 ± 0.01 W/kg, *p* < 0.001). The reduction in minimal knee angle (− 0.3 ± 0.1 degrees, *p* = 0.034), and increment in external knee extension moment in stance (− 0.01 ± 0.01 Nm/kg, *p* = 0.016) were small, although all stiffness’ substantially affected knee angle and knee moment compared to shoes only. No effect of stiffness on AFO generated power was found (*p* = 0.900).

**Conclusions:**

The optimal efficient DLS-AFO stiffness varied largely between persons with non-spastic calf muscle weakness. Results indicate this is caused by an individual trade-off between ankle angle and ankle power affected differently by AFO stiffness. We therefore recommend that the AFO stiffness should be individually optimized to best improve gait.

**Trial registration number:**

Nederlands Trial Register 5170. Registration date: May 7th 2015. http://www.trialregister.nl/trialreg/admin/rctview.asp?TC=5170

## Introduction

Persons with neuromuscular disorders and subsequent non-spastic calf muscle weakness are often limited in their daily physical activities [1, 2] due to an increased walking energy cost [3–5], caused by deviations in their gait pattern. Common gait deviations that lead to an increased walking energy cost are excessive ankle dorsiflexion and persistent knee flexion during stance, and reduced ankle power during push-off [6], which reduces walking speed [3, 5, 7].

To improve gait and decrease the elevated walking energy cost, persons with calf muscle weakness can be provided with a variety of passive dorsiflexion restrictive ankle-foot orthosis (AFO) types [7, 8]. The aim of these AFOs is to restrain excessive maximal ankle dorsiflexion and persistent knee flexion by providing a plantar flexion moment when the ankle moves into dorsiflexion during late stance [9, 10]. AFO types that also hold spring-like properties, such as carbon fiber dorsal leaf spring AFOs (DLS-AFO), can additionally support ankle power by storing energy during the stance phase when the ankle moves into dorsiflexion and releasing this energy during push-off when the ankle moves towards plantar flexion [11, 12]. Furthermore, plantarflexion is not completely restricted by DLS-AFOs, which may increase the ankle power further in patients with some remaining calf muscle strength. This increased ankle power may help initiate the swing phase, which, consequently, reduces walking energy cost compared to AFOs without spring-like properties [11–15].

Model simulations [16] and small studies in healthy subjects [17] and neurological patients [18] suggested that the effectiveness of spring-like AFOs to improve gait and reduce walking energy cost is stiffness dependent [16–18]. In neurological patients with calf muscle weakness, stiffer DLS-AFOs were more effective in reducing maximal ankle dorsiflexion [18, 19], although at the penalty of a reduction in push-off power at the ankle [18]. With regard to reducing walking energy cost, a moderate AFO stiffness appeared most beneficial [18], which was also reported in healthy subjects walking with an unpowered exoskeleton [17] and in simulation studies [16].

In neuromuscular disorders, however, the characteristics that affect the required stiffness to maximally improve gait, such as remaining muscle strength, weight and walking speed vary largely between patients. Hence, it is expected that the stiffness which optimally reduces the walking energy cost is patient dependent, as was indicated by a pilot study in polio patients with calf muscle weakness [20], but has not yet been studied in a large heterogeneous sample. We hypothesize that there will be variation in the most efficient DLS-AFO stiffness, which is expected to be a balance between normalizing ankle and knee kinematics and the ability to generate ankle power and the amount of energy returned (power generated) by the AFO. We therefore addressed the following research questions: 1) what is the effect of varying the stiffness of a DLS-AFO on walking energy cost, walking speed, gait biomechanics and AFO generated power? And 2) does the most efficient DLS-AFO stiffness for maximally reducing walking energy cost vary between persons?

## Methods

### Participants

Data used in this study were collected in the context of the PROOF-AFO trial, which has been described previously [21]. Participants in the PROOF-AFO trial were recruited from 12 hospitals and rehabilitation centers throughout the Netherlands and through the Dutch patient organization of neuromuscular diseases between July 2015 and July 2017. Individuals aged 18 years and older with non-spastic calf muscle weakness (unilateral or bilateral) were included. Other inclusion criteria were: using an AFO or reinforced orthopedic shoes for lower limb muscle weakness; able to walk for at least 6 min, if necessary with an assistive device; and weight below 120 kg as this was the maximum weight allowance of the intervention AFO. Patients were excluded when they had an indication for a knee-ankle foot orthosis or had pes equinus during weight bearing.

The study protocol of the PROOF-AFO trial was approved by the medical ethics committee of the Academic Medical Center (AMC) in Amsterdam, The Netherlands, and is registered at the Dutch trial register with number NTR5170. Before inclusion, all participants gave written informed consent.

### Ankle foot orthosis intervention

**Fig. 1:**
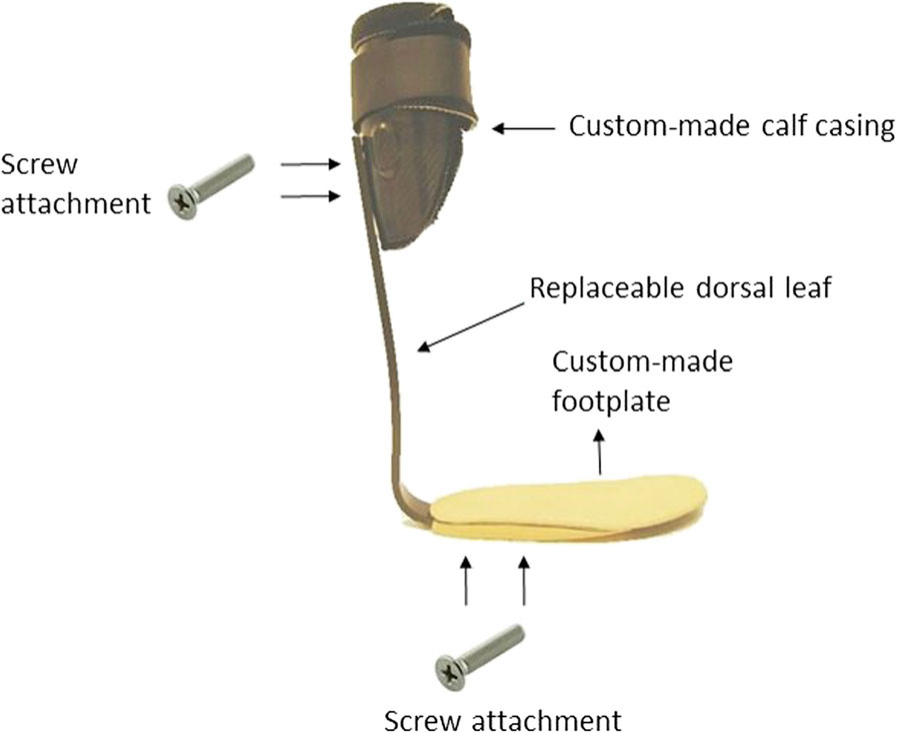
The intervention ankle foot orthosis with replaceable dorsal leaf spring

At study entry, participants were provided with a custom-made carbon spring-like AFO of which the stiffness could be varied (OIM orthopedietechniek, Noordwijkerhout, The Netherlands) (Fig. 1). The AFO consisted of a semi-stiff full-length footplate, a calf casing, and a replaceable carbon fiber dorsal leaf (Carbon Ankle Seven, Ottobock, Duderstadt, Germany) that could be mounted between footplate and calf casing with screws. We used five different carbon Ankle Seven leaves with stiffness levels ranging from flexible (K1) to stiff (K5). For each participant, the AFO stiffness of the 5 configurations were measured.

### Procedures

The AFO was fitted in normal shoes if possible, otherwise participants were provided with custom-made shoes. The alignment of the AFO was visually checked to make sure that during standing the knees were in neutral position (e.g. no hyperextension or knee flexion). When needed, heel height was adapted by placing cork heel wedges underneath the AFO.

Participants could get used to the intervention AFO by walking up and down a hallway with the first stiffness to be measured, with rail support if necessary. After participants felt comfortable walking with the AFO without rail support, six different conditions were tested: walking with shoes only and walking with AFO for 5 different stiffness configurations. For each condition, we measured walking energy cost at comfortable speed and gait biomechanics. The order in which the conditions were tested was randomly assigned using the “rand” function from Matlab (Matlab 2015, The Mathworks, Natick, USA). To make the effort manageable for the participant, walking energy cost and gait biomechanics were assessed on separate days one week apart, whereby walking energy cost was always assessed on the first measuring day. Measurements were performed at the gait lab of the department of Rehabilitation of the AMC in Amsterdam, The Netherlands.

### Measurements

#### AFO stiffness

AFO ankle and forefoot stiffness were measured with the Bi-articular Reciprocal Universal Compliance Estimator (BRUCE), which can measure the AFO stiffness reliable and with very small errors (0.02 Nm/degree) [22]. For each configuration, the AFO was strapped within the device and its rotational axis was aligned with the rotational axis of the BRUCE. Thereafter, to measure ankle stiffness, the AFO was moved into dorsiflexion and slowly released toward plantarflexion, each repeated three times, while ankle angle and exerted external moment were measured [22]. Forefoot stiffness was measured by aligning the foot of the BRUCE with the metatarsal heads. Thereafter, the footplate was moved towards dorsiflexion three times while toe angle and exerted external moment were measured.

#### Walking energy cost and walking speed

Walking energy cost at self-selected comfortable speed was measured during a 6-min walk test with simultaneous breath-by-breath assessment of oxygen consumption (VO_2_) and carbon dioxide production (VCO_2_) (Cosmed K4B^2^,Rome, Italy). The test was conducted on a 35-m oval track and participants were allowed to use their customary assistive device, e.g. crutch or cane, if necessary, which they then used throughout all stiffness conditions. Participants were not allowed to eat or drink sugar-holding beverages in the 90 min before the test. In between test conditions, participants had at least 10 min of rest during which the AFO stiffness was changed.

#### Gait biomechanics

Gait biomechanics was assessed with a 8-camera 100 Hz Vicon MX 1.3 system (VICON, Oxford, UK) while the participant was walking over a 12-m walkway with integrated force plates (1000 Hz, OR6–7, AMTI, Watertown, USA) at comfortable speed using the PlugInGait model. Two additional markers were placed in line with each other on the calf casing of the AFO to measure AFO deflection angle. Measurements for each condition were repeated until three valid trials for both legs were recorded (i.e. foot placed completely within the force plate and markers visible from heel strike on the force plate to ipsilateral heel strike).

### Data analysis

#### AFO stiffness

For each of the five AFO stiffness configurations, the recorded ankle and toe angle and exerted external moment measured with BRUCE, were plotted with a custom-written Matlab script (The Mathworks, Natick, USA). Ankle stiffness and forefoot stiffness (in Nm/degree) were then calculated by dividing change in external moment by change in ankle and toe angle, respectively [23].

#### Walking energy cost and walking speed

A period of at least 60 s within the last 3 min of the walking test wherein VO_2_ and VCO_2_ were in steady state was determined. For this period, the mean energy consumption (in J/kg/sec) (calculated as ((4.940* (VO_2_/VCO_2_) + 16.040)*VO_2_)) and walking speed (in m/s) were determined. To account for differences in walking speed between conditions and subjects, energy consumption was divided by walking speed to calculate the walking energy cost (in J/kg/m) [24].

#### Gait biomechanics

For each valid trial, the moments of heel-contact and toe-off were determined using the force-plate data and recorded videos. Thereafter, 3D data were processed using VICON Nexus (VICON, Oxford, UK) and spatiotemporal parameters as well as ankle, knee and hip kinematics and kinetics were retrieved. Using Matlab (The Mathworks, Natick, USA), these data were time normalized (0–100% of the gait cycles) and averaged across the three valid trials. Subsequently, specific gait outcomes that are considered relevant in the evaluation of AFOs were calculated [7], including (but not limited to) maximal dorsiflexion angle, maximal plantar flexion moment, peak ankle power, minimal knee angle and maximal external knee extension moment, all during stance.

In addition, AFO generated power was calculated to assess the contribution of the AFO to the ankle power. To calculate the AFO power, we assumed that the AFO deflection angle and AFO moment were zero as the AFO is in its neutral position during the swing phase. The AFO deflection angle could then be determined by calculating the change in relative position of the markers on the AFO, which can be different from the change in ankle angle as the leg might move relative to the AFO. The contribution of the AFO to the ankle moment was calculated by multiplying the AFO deflection angle with the AFO stiffness measured with BRUCE. Subsequently, AFO power was calculated by multiplying the AFO angular velocity, with the AFO moment.

### Statistics

Descriptive statistics were used to present social-demographic and disease characteristics of the study population and the study outcomes for each measured condition.

To determine the average effect of AFO stiffness on walking energy cost and speed, a repeated measures ANOVA was used. Post-hoc analysis consisted of pairwise comparisons with Bonferroni-corrections. The effect of AFO stiffness on gait biomechanics, and its contribution to ankle moment and power generation was determined with a multilevel linear mixed model with 3 levels; participant (third level), leg (second level), and stiffness configuration (first level). The multilevel analysis was chosen to account for the dependence between the measurements of the legs of the bilaterally affected persons. The effect of AFO stiffness, put in as absolute value, was modelled with a random intercept and random slope to account for the individual variance between individuals and legs. Spatiotemporal parameters were presented as the mean for all AFO legs.

To evaluate at individual level whether the most efficient AFO stiffness differed between persons, the most and least effective AFO in reducing walking energy cost were determined in each individual participant. The stiffness that reduced energy cost the most was considered the most efficient AFO. Patient characteristics between groups with a different most efficient AFO were tested with One-way ANOVA. Furthermore, to determine if an individual optimal stiffness is more efficient compared to a general one-size fits all optimal stiffness, the most efficient AFO was compared to the AFO stiffness that was on average (on group level) the most efficient, and shoes only. In addition, the speeds associated with the most and least efficient stiffness were compared. With Pearson correlations, the relation between differences in peak ankle power and maximal external knee extension moment and the difference in energy cost between the most and least effective AFO stiffness as well as the difference in these parameters between most efficient AFO and shoes only were tested. For bilateral patients, the mean delta of the two legs was used in this analysis.

## Results

### Subject characteristics

In total, 38 persons (21 males) with various neuromuscular disorders were included in the PROOF-AFO trial, of whom 1 was excluded as his quadriceps weakness progressed and a knee-ankle-foot orthosis was indicated. Social-demographic and disease characteristics of the remaining 37 participants are presented in Table 1.

**Table 1:** Baseline participant characteristics

Age in years	56.9 ± 15.5
Sex (m/f)	21/16
Height in cm	178 ± 10
Weight in kg	85.6 ± 16.2
Unilateral/bilateral affected	12/25
MRC plantar flexion of legs with AFO^1^	3 [2–4]
MRC sum score^a^	71.5 [64.8–75.5]
Diagnosis	Charcot-Marie-Tooth (*n* = 16) Poliomyelitis (*n* = 8) Nerve injuries (*n* = 9)^b^ Myotonic dystrophy (*n* = 2) Myoshi distal myopathy (*n* = 1) CIDP (*n* = 1) ^c^

^a^presented as median [inter-quartile range]

^b^Nerve injuries consisted of Radiculopathy (*n* = 2), Spinal disc herniation (*n* = 2), Spinal stenosis (*n* = 2), peroneal nerve injury (*n* = 1), partial cauda syndrome(*n* = 1) and partial paraplegia (*n* = 1)

^c^CIDP chronic inflammatory demyelinating polyneuropathy

### AFO stiffness

The mean ± SD ankle stiffness was 2.8 ± 0.4 Nm/degree for K1, 3.5 ± 0.4 for K2, 4.3 ± 0.5 for K3, 5.3 ± 0.7 for K4 and 6.6 ± 1.1 for K5. The mean ± SD footplate stiffness was 0.2 ± 0.1 Nm/degree.

### Effect of AFO stiffness on gait

#### Walking energy cost and walking speed

Due to a technical error in the data for one participant with AFO stiffness K5 while assessing walking energy cost, data of only 36 persons could be used in the analysis of this outcome.

**Fig. 2:**
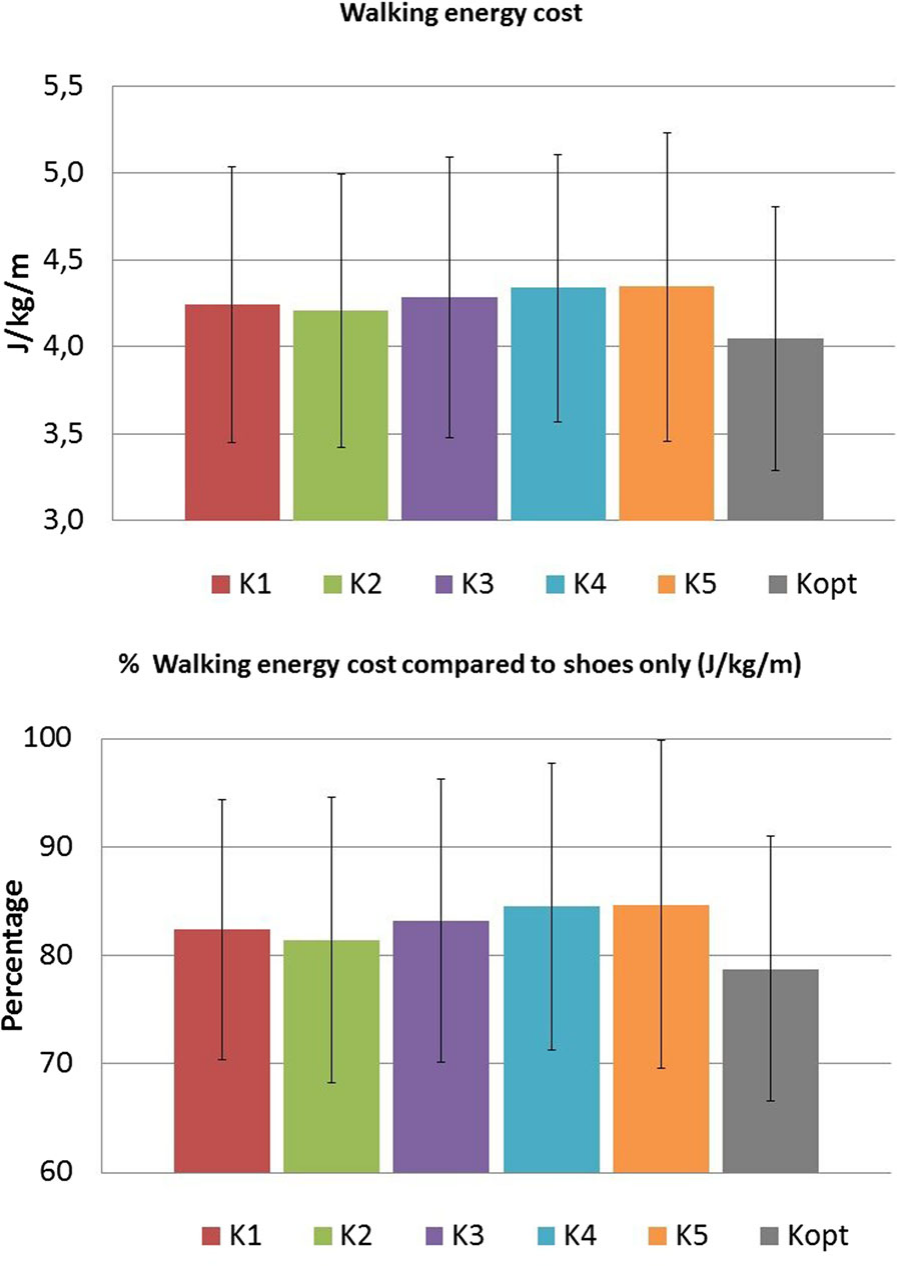
Percentage of walking energy cost when walking with AFO compared to shoes only, where 100% represents shoes only. K = stiffness. Kopt = most efficient AFO stiffness

On group level, for all AFO stiffness configurations, walking energy cost was lower compared to shoes-only (shoes only: 5.23 ± 1.15 J/kg/m, smallest reduction K5: -0.88 ± 0.87 J/kg/m (− 15.3%), largest reduction K2: -1.02 ± 0.80 J/kg/m (− 18.6%)), with no significant differences found between AFO stiffness configurations (F = 2.60, *p* = 0.059) (Fig. 2).

Walking speed was higher compared to shoes-only for all AFO stiffness configurations (shoes only: 0.87 ± 0.21 m/s, smallest improvement K5: + 0.17 ± 0.17 m/s (+ 19.5%), largest improvement K2: + 0.21 ± 0.17 m/s (+ 24.1%)), with significant differences found between AFO stiffness’ (F = 3.75, *p* = 0.013). Post-hoc analysis revealed that walking speed was significantly higher in K2 compared to K5 (+ 0.04 m/s (+ 3.7%)).

#### Gait biomechanics

**Fig. 3:**
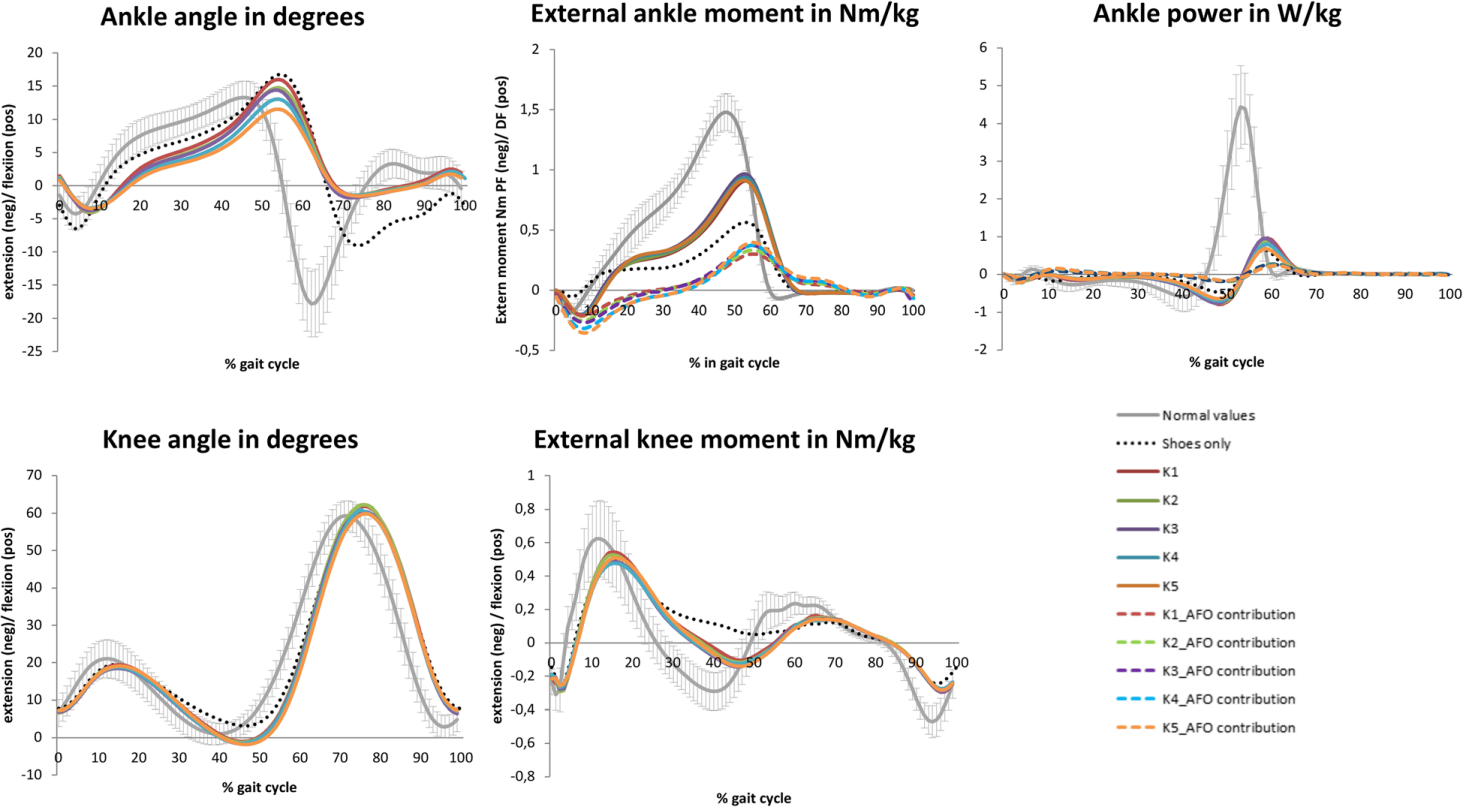
Effect of AFO stiffness on ankle and knee biomechanics

With each increase of 1 Nm/degree of AFO stiffness, maximal dorsiflexion angle reduced significantly by 1.1 degree and peak ankle power by 0.09 W/kg (Table 2). Regarding knee biomechanics, although all stiffness levels substantially improved knee angle and knee moment compared to shoes only (Fig. 3), increasing stiffness with 1 Nm/ degree reduced the minimal knee angle significantly with 0.3 degrees and increased the external extension moment by 0.01 Nm (Fig. 3). Mean values for all stiffness levels can be found in the Additional file 1: Table S1.

**Table 2:** Effect of AFO stiffness on biomechanical gait parameters

Outcome parameter	β0 intercept (S.E)	β1 effect stiffness (S.E.)	*p-*value
*Model:* β0 + β1*stiffness (Nm)			
*Gait parameters*			
Maximal dorsiflexion angle	20.23 (0.95)	- 1.048 (0.137)	< 0.001
Peak ankle power (biological + AFO power)	1.83 (0.109)	−0.093 (0.013)	< 0.001
Minimal knee angle	−0.59 (1.10)	−0.267 (0.126)	0.034
Maximal knee extension moment	−0.14 (0.03)	−0.013 (0.006)	0.030
*AFO contribution*			
Maximal power AFO	0.48 (0.04)	−0.001 (0.008)	0.900

Due to processing problems with the markers on the AFO (*n* = 5) and persons using a walking stick during the gait analysis (*n* = 3), the AFOs’ contribution to the power generation was determined for 29 persons. No effect of AFO stiffness on the AFO generated power was found (Table 2 and Fig. 3).

### Individual most efficient AFO stiffness

The individual most efficient AFO stiffness differed between persons, with stiffness 3 (K3) most frequently being the most efficient (*n* = 11). The other stiffness’ were most efficient for: K1 in 8 persons, K2 in 6 persons, K4 in 5 persons and K5 in 6 persons. The least efficient AFO stiffness was most frequently K5 (14 persons) and K4 (in 12 persons).

While the individual least efficient AFO stiffness reduced walking energy cost with 0.70 ± 0.84 J/kg/m (−13.2%) compared to shoes only, the individual most efficient AFO stiffness reduced it by an additional 0.49 J/kg/m (energy cost most efficient AFO: 4.05 ± 0.76 versus least efficient AFO: 4.54 ± 0.84 J/kg/m), This additional reduction of 10.7% showed large inter-individual variation (range − 3.7% to − 24.0%). Walking speed was 0.04 ± 0.06 m/s higher with the most efficient AFO stiffness compared to the least efficient stiffness (1.09 ± 0.17 versus 1.05 ± 0.18 m/s), with a mean increase of 4.4% (range: − 11.8 to + 13.9%).

The individual most efficient AFO stiffness had a significant lower energy cost compared to the general most efficient stiffness, which was K2, (4.05 ± 0.76 J/kg/m versus 4.21 ± 0.79 J/kg/m, *p* < 0.001), with no differences found for walking speed (1.09 ± 0.17 versus 1.08 ± 0.17 m/s, *p* = 0.559).

**Fig. 4:**
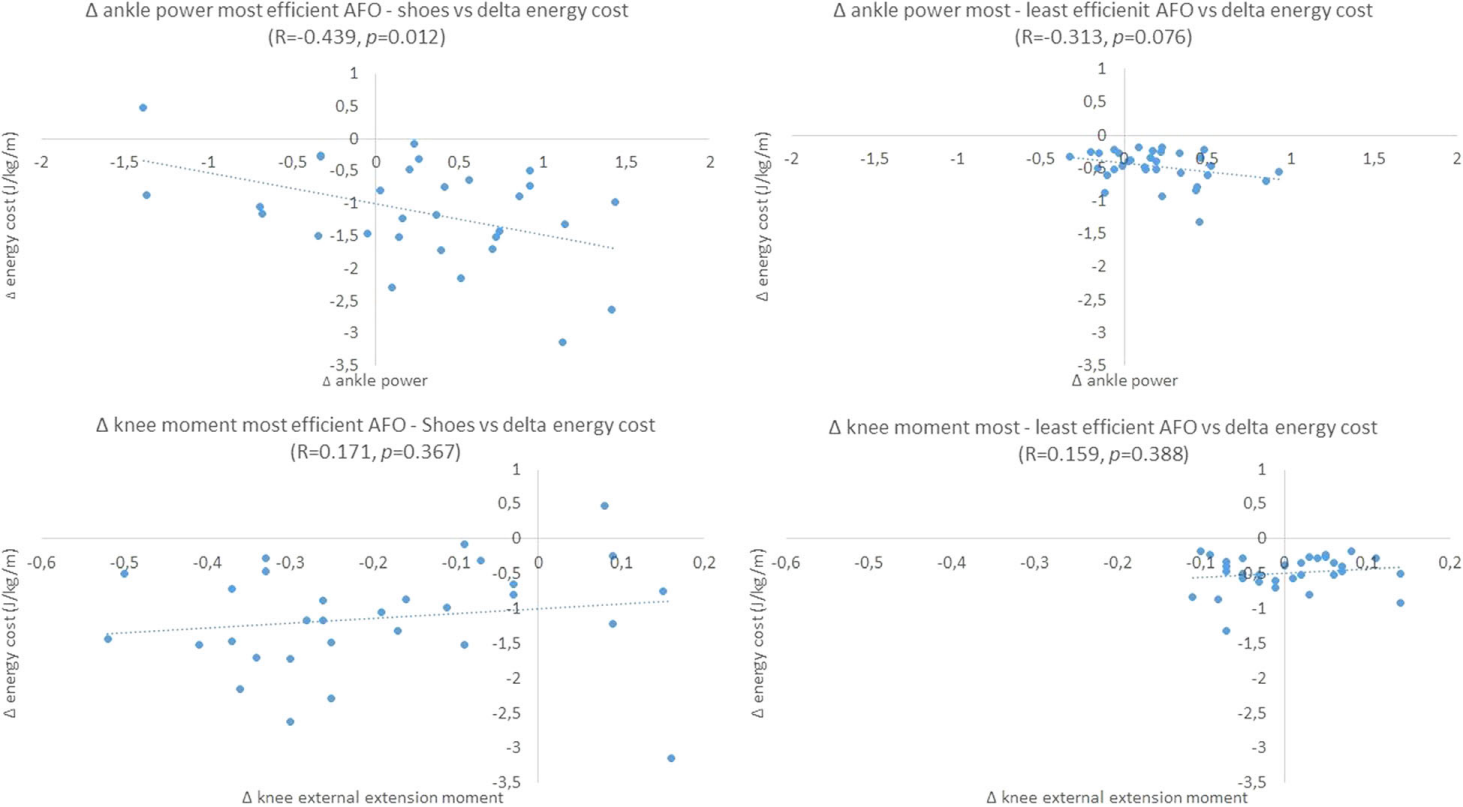
Relation between reduction in energy cost and change in ankle power and knee moment, respectively, for most efficient AFO versus shoe only (left panels) and most efficient AFO versus least efficient AFO (right panels). Kopt = most efficient AFO stiffness; Kleast = least efficient AFO stiffness. Negative delta in energy cost means an improvement. Positive delta in ankle power means an increase in ankle power. Negative delta in knee external moment means an increase in external knee extension moment

Increase in peak ankle power with the most efficient AFO stiffness compared to shoes-only was significantly related with reduction in energy cost (r = − 0.439, *p* = 0.012), while increase in external knee extension moment was not (r = 0.171, *p* = 0.367) (Fig. 4). Difference in peak ankle power and external knee extension moment between the most and least efficient stiffness were both not significantly correlated with the difference in energy cost between the most and least efficient stiffness (ankle power: r = − 0.313, *p* = 0.076; knee moment: r = 0.159, *p* = 0.388).

## Discussion

Although all AFO stiffness levels considerably improved walking energy cost between 15 and 19% and speed between 20 and 24% compared to shoes only, individually optimizing the AFO stiffness improved these parameters further. Namely, the individual optimal AFO stiffness reduced walking energy cost with an additional 10.7% at an increased speed of 4% compared to the least efficient AFO stiffness. Furthermore, the individual most efficient AFO stiffness reduced walking energy cost 4% beyond the effect of the general optimal efficient AFO stiffness on group level. In addition we showed that increasing AFO stiffness had conflicting effects on ankle angle and ankle power while it only mildly effected the knee biomechanics, which can be used to individually tune the gait biomechanics.

AFO stiffness variation reduced the walking energy cost considerably at the individual level, while on group level no effect of stiffness was found. This was also reported in cerebral palsy patients [14, 25], but is in contrast with small studies in healthy subjects [17] and a homogenous sample of stroke and MS patients [18]. As healthy subjects walk biomechanically comparable, and the stroke patients all were unilaterally affected, had some spasticity and good balance, it can be expected that there is limited variation in the most efficient stiffness within these investigated populations despite differences in body mass [17, 18]. In our study, a heterogeneous population with regard to diagnosis, muscle strength and sensory problems was included, which all influence gait and apparently the most efficient AFO stiffness. When evaluating the effects on individual level, the most efficient AFO stiffness, compared to the least efficient stiffness, had on average a 11% lower walking energy cost. This is considered a highly clinical relevant and meaningful effect, as it is larger than the smallest detectable change [3] and providing non-optimized AFOs to polio patients with calf muscle weakness merely reduced walking energy cost by 7% [7]. Contrary to energy cost, an effect for walking speed on group level was found, and an AFO stiffness between 2.8 and 4.3 Nm/degree resulted in the highest speed (Fig. 2), which was also seen in patients with stroke and MS [17, 18, 26]. However, in 30% of the subjects, the most efficient AFO had a stiffness higher than 4.3 Nm/degree, without negatively influencing the walking speed in these people, again indicating the relevance of individualising AFO stiffness.

The optimal AFO stiffness for the reduction in energy cost was hypothesized to be a trade-off between sufficient stiffness to normalize ankle and knee kinematics and flexibility to be able to generate ankle power. Regarding knee flexion and knee moment, AFO stiffness only had a small and probably clinically irrelevant effect as the lowest stiffness tested made the knee already extend sufficiently. It seems that when the AFO reaches a certain stiffness the knee extends, and since additional knee extension may anatomically not be possible, additional effects on knee extension are limited [14].

Regarding the ankle biomechanics, although on average the lower stiffness levels (e.g. K1 and K2) already normalized the maximal ankle dorsiflexion angle in our patients, some individuals required a more substantial stiffness to reduce the maximal ankle dorsiflexion to reference levels. This was at the cost of the ability to generate ankle power. With increasing AFO stiffness, maximal ankle dorsiflexion reduced with each additional 1 Nm/degree of stiffness by approximately 1 degree, which is in line with previous studies in lower limb impairments, stroke and MS patients [19, 27, 28] and may be used to individually tune the stiffness to normalize the maximal ankle dorsiflexion angle. However, the reduced range of motion reduces the ability to generate ankle power, as previously indicated [13, 14]. As a consequence of the reduced maximal dorsiflexion angle, the Achilles-tendon is less stretched and less energy is stored during the stance phase. The energy storage in the Achilles-tendon is further reduced by the fact that the AFO takes over part of the plantarflexion moment normally generated by the calf muscles, thereby reducing the force on the tendon and, consequently, the energy storage and recoil of the Achilles-tendon [29]. This reduced energy recoil may explain the reduced ankle power, as the AFO generated power does not change with increasing stiffness. In addition, a higher AFO stiffness also causes a higher resistance towards plantarflexion during push-off which limits the ankle power generation in patients who are able to generate push-off power actively. However, as none of our patients were able to perform a standing heel-rise, this effect is probably negligible.

Furthermore, it is noteworthy that the AFO does not normalize the ankle angle during mid-stance. Although the center-of-pressure moves more forward compared to shoes only, as indicated by the increment in external ankle moment during this phase, the progression is still behind what is seen in healthy individuals. It might be that patients need specific training to move the center-of-progression further forward during mid-stance and consequently normalize their ankle angle in mid-stance.

The conflicting effects of an increase in AFO stiffness on ankle biomechanics and differences in patient characteristics can explain the large variation in most efficient stiffness and the absence of a relation between changes in ankle power or knee moment and energy cost between stiffness levels. Some patients need a higher stiffness level, at the disadvantage of ankle power, to normalize the maximal ankle dorsiflexion and external knee extension moment, which also reduces the energy cost [30, 31]. In others, a low stiffness sufficiently reduces the ankle angle and higher stiffness levels are not warranted because of the negative effects on ankle power [32]. Although we did not find differences in characteristics between groups with a different optimal AFO stiffness, we hypothesize that variation in individual characteristics, which determine the required AFO stiffness to normalize the joint angles and moments, such as walking speed, remaining strength and weight, explain partly which AFO stiffness is most optimal [16]. As in patients it is impossible to systematically vary these characteristics, simulation models should be used to evaluate and objectify the effect of these characteristics on the optimal AFO stiffness.

When interpreting our results between the most and least efficient AFO, the measurement error for walking energy cost has to be considered. The 10.7% effect of stiffness variation we found, is larger than the smallest detectable difference for energy cost reported in poliomyelitis (9.4%) [3] and cerebral palsy patients (6.8%) [33]. Furthermore, it is also larger than the effect of stiffness optimization in healthy subjects, where the observed 7% reduction in energy cost was considered equivalent to the effect of unloading a 4 kg backpack for an average person [17]. Therefore, we are confident that the effects found at individual level are substantial and clinically relevant. However, this rather large variability might have influenced which AFO stiffness was most efficient for specific subjects. Consequently, the correlation between change in walking energy cost and change in gait biomechanics might be underestimated. A second consideration is that our range of tested stiffness levels does not cover the full range of AFO stiffness levels provided in usual orthotic care as the lowest stiffness tested is already stiffer compared to common types of usual care AFOs, while our stiffest AFO cannot be considered rigid. Inclusion of a wider range of stiffness levels was not feasible as dorsal leafs with a lower stiffness differed considerably in width and thickness, and consequently could not be fitted within the modular AFO. The limited stiffness range applied reduced our group effect sizes of stiffness on walking energy cost and walking speed, while it also limits the generalizability. However, as the most efficient stiffness was often not K1 or K5, it can be concluded that the most efficient AFO stiffness was included within the tested stiffness range for most of the patients. With regard to the gait biomechanics, we analysed only 3 steps per person which may have influenced our results. Although the number of steps is relatively low, this is a common number in this population [7, 14].

## Conclusions

We conclude that in patients with neuromuscular disorders and non-spastic with calf muscle weakness, the optimal AFO stiffness for the reduction of walking energy cost varies largely between individuals. The individual optimal efficient AFO stiffness reduced the energy cost with a clinically relevant 21% compared to shoes only and with 10.7% compared to the least efficient AFO stiffness. In addition, the individual optimal efficient AFO stiffness was more effective compared to a general, one-size fits all optimal efficient stiffness. As an increase in AFO stiffness reduced the maximal ankle dorsiflexion angle but negatively affected the ankle power, the optimal AFO stiffness to normalize gait is an individual trade-off between these gait biomechanics and subject characteristics. Therefore, we recommend that in non-spastic calf muscle weakness, the AFO stiffness should be individually optimised as this results in the lowest walking energy cost [34].

## Supplementary information

**Additional file 1:** Table S1. Gait and mechanical outcomes for the 5 different stiffness configurations.
