# Supplementary material for: Modifying ankle foot orthosis stiffness in patients with calf muscle weakness: gait responses on group and individual level
Source: J Neuroeng Rehabil. 2019 Oct 17;16:120. doi: 10.1186/s12984-019-0600-2 (PMC6798503; doi:10.1186/s12984-019-0600-2)
Supplement: Supplementary file 1 — Additional file 1: Table S1. Gait and mechanical outcomes for the 5 different stiffness configurations. [file 12984_2019_600_MOESM1_ESM.docx]

**Supplementary table. Gait and mechanical outcomes for the 5 different stiffness configurations**

|  | **K1** | **K2** | **K3** | **K4** | **K5** |
| --- | --- | --- | --- | --- | --- |
| **Spatiotemporal** | | | | | |
| **Walking energy cost** (J/kg/m) | 4.24±0.13 | 4.21±0.13 | 4.29±0.14 | 4.34±0.13 | 4.35±0.15 |
| **Walking speed** (m/s) | 1.08±0.17 | 1.08±0.17 | 1.07±0.19 | 1.05±0.19 | 1.04±0.17 |
| **Cadence** (steps/min) | 101±10 | 101±9 | 102±9 | 101±10 | 100±10 |
| **Stride length** (m) | 1.25±0.19 | 1.22±0.21 | 1.24±0.2 | 1.24±0.20 | 1.22±0.19 |
| **Timing toe-off**  (% gait cycle) | 65.8±2.8 | 65.7±2.7 | 65.8±3.0 | 65.6±3.2 | 65.8±3.0 |
| **Timing heel-off**  (% gait cycle) | 49.4±7.0 | 48.8±7.0 | 47.8±7.4 | 46.8±7.7 | 46.6±7.8 |
| **Joint Angles** | | | | | |
| **Maximal ankle angle in stance** | 17.3±5.1 | 16.0±5.2 | 14.7±4.7 | 14.3±4.5 | 12.8±4.7 |
| **Minimal knee angle in stance** | -1.7±6.6 | -2.0±6.1 | -2.2±6.8 | -2.4±5.8 | -2.7±6.1 |
| **Joint Moments** | | | | | |
| **Maximal ankle moment** (Nm/kg) | 1.02±0.25 | 1.04±0.23 | 1.06±0.25 | 1.06±0.24 | 1.03±0.25 |
| **Maximal AFO moment** (Nm/kg) | 0.35±0.10 | 0.39±0.10 | 0.44±0.13 | 0.47±0.14 | 0.49±0.17 |
| **Maximal knee extension moment**  (Nm/kg) | -0.17±0.19 | -0.18±0.019 | -0.21±0.21 | -0.20±0.21 | -0.22±0.21 |
| **Joint Powers** | | | | | |
| **Peak ankle power**  (W/kg) | 1.54±0.53 | 1.48±0.55 | 1.42±0.51 | 1.30±0.51 | 1.14±0.51 |
| **Peak AFO generated power** (W/kg) | 0.48±0.23 | 0.49±0.23 | 0.47±0.22 | 0.47±0.27 | 0.43±0.27 |
| **Timing peak power**  (% gait cycle) | 59.5±2.6 | 59.5±2.7 | 59.2±2.5 | 59.5±2.7 | 59.7±2.9 |
| **Mechanical joint work*** | | | | | |
| **Hip Positive work**  (J/kg/m) | 0.34±0.08 | 0.34±0.09 | 0.34±0.08 | 0.34±0.08 | 0.34±0.08 |
| **Knee Positive work**  (J/kg/m) | 0.15±0.08 | 0.15±0.09 | 0.13±0.07 | 0.14±0.10 | 0.14±0.07 |
| **Ankle Positive work**  (J/kg/m) | 0.11±0.04 | 0.11±0.03 | 0.10±0.03 | 0.10±0.03 | 0.08±0.03 |
| **AFO Positive work**  (J/kg/m) | 0.06±0.02 | 0.06±0.02 | 0.06±0.02 | 0.06±0.03 | 0.06±0.03 |

* calculated as the integral of the respective power curves
